# Preliminary Effects of Osteopathic Manipulative Medicine on Reactive Oxygen Species in Parkinson’s Disease: A Randomized Controlled Pilot Study

**DOI:** 10.7759/cureus.31504

**Published:** 2022-11-14

**Authors:** James Docherty, Joerg R Leheste, Jayme Mancini, Sheldon Yao

**Affiliations:** 1 Family Medicine, United Health Services, Johnson City, USA; 2 Biomedical Sciences, New York Institute of Technology College of Osteopathic Medicine, Old Westbury, USA; 3 Osteopathic Manipulative Medicine, New York Institute of Technology College of Osteopathic Medicine, Old Westbury, USA

**Keywords:** parkinsons, reactive oxygen metabolite, osteopathic manipulative medicine (omm), osteopathic manipulative treatment (omt), pd, osteopath, reactive oxygen species, parkinson' s disease

## Abstract

Context

Parkinson’s disease (PD) is the second most common neurodegenerative disorder and causes many clinical manifestations including bradykinesia, tremor, postural instability, and musculoskeletal stiffness. Neurodegeneration is commonly associated with oxidative stress. Oxidative stress has not been measured in PD in relation to the manual techniques used in Osteopathic Manipulative Treatment (OMT).

Objective

To investigate the effect of OMT on oxidative stress biomarkers in PD.

Methods

In this randomized non-blinded study, 32 PD subjects were separated by block randomization into counseling and OMT groups, receiving respective interventions twice a week for six weeks. The counseling arm received informative sessions while the OMT arm received a set treatment protocol. Biomarkers of oxidative stress, malondialdehyde (MDA), dityrosine (DT), 3-nitrotyrosine (3-NT), 8-hydroxy-2-deoxyguanosine (8-OHdG), and 8-isoprostane were measured before and after the first session and at weeks three, six, and 10 (four weeks after conclusion of intervention).

Results

No significant changes were found in blood plasma levels of MDA, DT, 3-NT, or 8-OHdG during or after intervention compared to controls (p > 0.05). No significant changes were found in urine 8-OHdG or 8-isoprostane during or after intervention compared to controls (p > 0.05).

Conclusion

OMT used in this study did not significantly affect the chosen oxidative stress biomarkers, however many limitations of the study may have impeded possible findings.

## Introduction

Parkinson’s disease (PD) is the most common progressive neurodegenerative movement disorder in humans. Globally, the prevalence of PD is seven to 10 million. The incidence of PD increases with age. In those diagnosed before age 50, it is at an estimated 4% in North America. Annually, 60,000 people in the United States are diagnosed with PD and the expected prevalence for 2030 is 1,238,000 [1,2]. Many factors contributing to disease progression are still unclear, however, age, genetics, and environmental factors are involved [3].

PD is a complex disease with variable signs and symptoms commonly including resting tremor, rigidity, bradykinesia and postural instability, which directly leads to diminished control of balance and a high rate of falling [4-6]. Despite numerous general advances in the understanding of neuroinflammation and cell death, management has been lagging behind with 70% of all PD individuals falling at least once and 50% falliing twice or more per year according to a small US study (56 participants) [7,8]. Previous studies suggest that manual treatments, including Osteopathic Manipulative Treatment (OMT), which is a form of non-invasive manual treatment, may be effective in improving gait, range of motion, and constipation in PD subjects, postural instability in healthy elderly subjects, gait asymmetry in healthy young males, and balance in subjects with dizziness [9-16]. Unfortunately, research on OMT as a potential treatment alternative to more invasive surgical or pharmaceutical has been lacking [17].

Neuroinflammation and oxidative stress are commonly linked with brain injury and neurodegeneration such as in PD [8,18,19]. High levels of oxidative stress, especially over a prolonged time, significantly exacerbate somatic and neuronal dysfunction. In this sense, oxidative stressors are not only the result of PD but also pathogenic contributors [20]. The production of the neurotransmitter dopamine (DA) produces abundant reactive oxygen species (ROS). DA neurons therefore tend to be more vulnerable to additional forms of oxidative stress which could explain their selective demise in PD [21]. Along the same paradigm, several of the genes tied to familial forms of PD (e.g. PRKN, SNCA, PINK1, DJ1 and LRRK2) are involved in the generation of oxidative stress [22]. Besides damage to nucleic acids and proteins, lipid peroxidation in the substantia nigra pars compacta (SNc) was found to be elevated 10-fold in human postmortem PD tissues causing oxidative cell damage [20,23-26]. The accumulation of damaged proteins, lipids, and nucleic acids is believed to ultimately trigger dopaminergic cell death in the SNc causing clinical manifestations of PD. In fact, mouse models of PD have shown significant increases in ROS in the midbrain and striatum, specifically L,L-dityrosine (DT) and 3-nitrotyrosine (3-NT) which is thought to cause direct cellular damage and death [27,28]. While the SNc remains the focal point of the disease, systemic oxidative stress and damage levels have been thoroughly documented [29-34]. In the cerebrospinal fluid (CSF), individuals with PD showed increased 8-hydroxy-2-deoxyguanosine (8-OHdG, oxidized DNA) [35-37]. A previously established biomarker of systemic oxidative stress used in PD is 8-isoprostane [36,38]. Molecular serum biomarkers of oxidative stress found elevated in PD are malondialdehyde (MDA, oxidized lipids), 8-OHdG, and 3-NT (protein oxidation), all of which are linked to disease progression [31,39-41].

Biomarkers are now frequently used to monitor disease states linked to elevated oxidative stress [42,43]. MDA, for example, has been shown to decrease in PD after resistance training, a therapy shown to be beneficial in the disease [44,45]. In addition, urinary 8-OHdG was elevated in PD, having a positive correlation with disease burden, and an inverse correlation with levodopa use, the hallmark of pharmaceutical PD therapy [46,47]. Molecular biomarkers with proven clinical validity could be useful in establishing OMT as an effective treatment option for PD-related oxidative stress. Assessing the effect of a single treatment session will enable us to distinguish potential acute and chronic treatment effects. When designing osteopathic clinical studies, it is critical to be aware of OMT-specific challenges such as the difficulty of blinding, especially in individuals who have previously received OMT. Other challenges are inter-physician variability in diagnosis and treatment of somatic dysfunction which could affect outcomes and their interpretability, as well as the placebo effect due to outcome expectations of OMT recipients [48-51].

The aim of the study was to determine if OMT was effective in changing oxidative stress levels as measured via established biomarkers. We hypothesize that MDA, DT, 8-OHdG, 3-NT, and 8-Isoprostane will significantly change with the application of 12 treatments of an established OMT sequence. Our scientific hypothesis is based on the observation that nigrostriatal circuits and DA neurons of the SNc are at heightened susceptibility to mitochondrial dysfunction and subsequent cell death. This is due to a naturally elevated baseline of ROS in DA neurons (catecholamine-driven Fenton reaction) and exacerbated by a local increase of iron, decrease of neuromelanin and enhanced Lewy body formation [52,53]. The mechanisms are not yet fully understood, but the systemic ROS burden in PD increases with the progression of the disease. Corroborating evidence has been provided for raised serum 8-OhdG, MDA, nitrite, and ferritin [54]. There is evidence suggesting that progressive muscle inactivity and sedentariness, as seen in PD, contribute significantly to the systemic ROS burden and muscle wasting of people with PD [55,56]. So far, OMT has revealed its greatest value in the management of motor-related symptoms such as gait, posture, and balance supporting this approach [16,17].

## Materials and methods

This non-blinded, randomized, controlled trial of a pre-defined OMT sequence targeting mobility and balance in PD was conducted at the clinic of New York Institute of Technology, a private, not-for-profit institution (Institutional Review Board approval: 975, registered at clinicaltrials.gov: NCT02107638). We recruited subjects from March 2015 through June 2019. Recruitment took place through an internet search (see clinicaltrials.gov), referrals by the in-house neurologist, personal advertisements at local PD support groups, and recruiting tables at local fundraising events for PD-associated charities and organizations.

Subjects

Inclusion criteria targeted individuals greater than ≥40 years of age with a diagnosis of PD by a neurologist according to the UK Parkinson’s Disease Society Brain Bank Clinical Diagnostic Criteria (UKPDBB) [57]. Additional inclusion criteria were: 1) Movement Disorder Society-Unified Parkinson’s Disease Rating Scale (MDS-UPDRS) Part III score of >30; 2) Sensory Organization Test (SOT) score of <75; or 3) Mini-Balance Evaluation Systems Test (Mini-BESTest) score of <19. Subjects who had a history of any other neurologic conditions or who were unable to complete the assessment tools were excluded. These criteria are based on validated measures of motor symptoms in moderate to severe PD and linked to elevated systemic ROS production [58-60].

Study design

Eligible and consenting individuals were randomly assigned by blocks of two (determined by date of study consent signing) into OMT or PD patient counseling groups. This was determined by a research coordinator who was not personally involved with the interventions. Each OMT treatment consisted of a 30-minute treatment sequence previously established and validated for use in motor dysfunction [15]. Treatment occurred twice per week for six weeks. The control group received weekly one-hour counseling sessions from a healthcare provider for six weeks controlling for social-placebo effects and time (Table 1) [15]. The OMT sequence was performed by one of four physicians board certified in OMT and according to physician availability. The counseling sessions were provided by the same group of physicians or a trained exercise physiologist certified in clinical nutrition.

**Table 1:** List of techniques used in the Park-OMT protocol and list of education sessions in the counseling protocol. Park-OMT: Parkinson's Disease Osteopathic Manipulative Treatment. PD: Parkinson's Disease. PT: Physical Therapy. OT: Occupational Therapy.

Park-OMT Protocol	Counseling Control Protocol
1. Suboccipital Release	1. Detailed History of Parkinson’s Disease, review of medications, exposures (risk factors), family history, quality of life
2. Compression of the Fourth Ventricle (CV-4)	2. Trauma counseling
3. Supine Cervical Spinal Articulation	3. Falls, including causes and precautions
4. Muscle Energy Technique of the Cervical Spine (Side bending and rotation)	4. Gait counseling
5. Bilateral Spencer’s Technique of the Shoulder	5. Freezing and balance Counseling
6. Muscle Energy Treatment of the Radial Head	6. Nutrition and PD part 1 of 2
7. Circumduction of the Wrist Bilaterally	7. Nutrition and PD part 2 of 2
8. Sacroiliac Joint Gapping Bilaterally	8. Exercise, including stretching, yoga, PT/OT basics, posture
9. Muscle Energy Technique of the Lower Extremity Adductor Muscles Bilaterally	9. Mental health and PD
10. Psoas Muscle Energy Technique Bilaterally	10. Genetics and PD
11. Muscle Energy Technique of the Hamstring Bilaterally	11. Wellness, relaxation, meditation, and overall health
12. Bilateral Ankle Articulatory Technique	12. Non-motor symptoms of PD
13. Muscle Energy Technique to the Plantar and Dorsiflexion muscles of the Ankle Bilaterally	13. Medication side effects
14. Seated Thoracic and Lumbar Spinal Articulation	14. Sleep hygiene
15. Seated Active Myofascial Stretch to the Thoracic Spine (side bending and rotation)	

Blood extraction, urine collection, and quantification

All subjects consented to blood draws after a 12-hour washout period from their PD medication on day one immediately before and after intervention (Pre and Post), week three (visit seven), week six (visit 12), and week 10 (four-week-washout). Phlebotomy tourniquets were applied for less than one minute and removed immediately upon establishing blood flow. All specimens were centrifuged at 1200 g for 15 minutes, and serum was portioned into multiple aliquots containing sufficient volume to provide for duplicate analysis for each assay and immediately frozen at -80° C. Urine samples were collected into standard urine collection vessels and frozen at -80° C. Serum samples were analyzed in duplicate on an enzyme-linked immunosorbent assay (ELISA) platform using the following assay kits from Abcam (Waltham, Boston, USA) and according to the manufacturer’s instructions: MDA (ab238537), 3-NT (ab113848), and 8-OHdG (ab201734) for oxidative damage. Dityrosine was analyzed using the Japan Institute for the Control of Aging (JaICA) kit KDT­010/E. Urine samples were analyzed in duplicate on an ELISA platform using the following Abcam assay kits: 8-OHdG (ab201734) and 8-isoprostane (ab175819); 8-OHdG and 8-isprostane. These values were then normalized against creatinine (ab65340).

Statistical analysis

Power analysis has been performed using G*Power 3.1.9.2 to estimate a reasonable sample size for the study. From the results in the preliminary studies (UPDRS, Mini-BESTest), effect sizes were estimated to be medium or large (Cohen’s d values range from 0.5 to 1.2). To achieve 80% of statistical power, a total sample size of 126 (64 in each group) was required to detect a medium effect size (Cohen’s d=0.5), and a total sample size of 24 (12 in each group) was required to detect a large effect size (Cohen’s d=1.2), α=0.05. Based on these determinants of power analysis and anticipated attrition of PD patients over 24 months, we recruited 32 PD subjects [61].

A repeated measures analysis of variance (ANOVA; α=0.05) using SPSS software version 25 (IBM Corp., Armonk, NY, USA), was conducted to determine whether biomarker levels had significantly responded to OMT.

The hypothesis that the mean levels of each biomarker were significantly different between the two groups was tested by a repeated measures ANOVA using SPSS software version 25, α=0.05.

## Results

Subject recruitment

**Figure 1:**
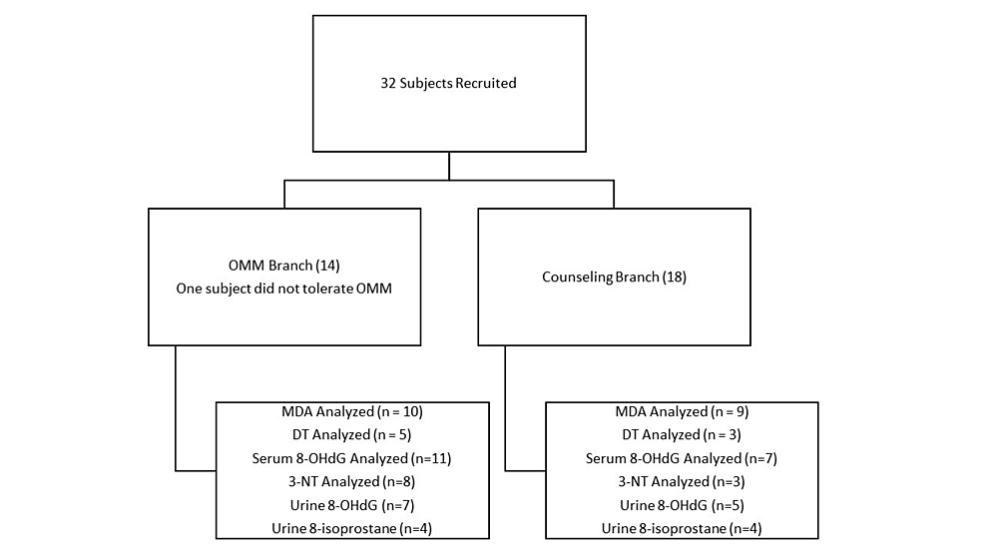
Randomization, Treatment Assignments, and Follow-up. Subjects were recruited from March 2015 through June 2019 from the Adele Smithers Parkinson’s Disease (PD) Treatment Center of the New York Institute of Technology College of Osteopathic Medicine. OMM: Osteopathic Manipulative Medicine. MDA: Malondialdehyde. DT: L,L-dityrosine. 8-OHdG: 8-hydroxy-2-deoxyguanosine. 3-NT: 3-nitrotyrosine.

This study includes a total of 32 subjects in alignment with the inclusion criteria (Figure 1). Fourteen were assigned to the OMT group. Eighteen were assigned to the counseling group. One subject did not tolerate OMT treatment and decided to withdraw. We ended recruitment when the budget for recruitment activities was exhausted. Aside from one subject unable to tolerate OMT due to discomfort, there were no other reported complications. In several instances, the volume of sample material turned out to be insufficient for analysis, decreasing the study’s subject pool to 19 (10 in the OMT group, nine in the counseling group). The OMT group consisted of 20% females and 80% males and an average age of 65.6 (±2.5) years with a diagnosis time of 6.4 (±1.6) years. The counseling group consisted of 44% females and 56% males and a mean age of 71.3 (±2.3) years with a diagnosis time of 7.8 (±2.3) years. Both groups ranked similarly regarding their PD-related functional disability with a mean Hoehn and Yahr (H&Y) score of 2.0 (±0.3) and 2.1 (±0.3) for the OMT and counseling groups, respectively. Baseline characteristics are summarized in Table 2.

**Table 2:** Subject characteristics. OMM: Osteopathic Manipulative Medicine.

Branch	Female (%)	Male (%)	Average Age (SE)	Average Years diagnosed (SE)	Average Hoehn and Yahr Score (SE)
OMM	2 (20%)	8 (80%)	65.6 (2.5)	6.4 (1.6)	2.0 (0.3)
Counseling	4 (44%)	5 (56%)	71.3 (2.3)	7.8 (2.3)	2.1 (0.3)

Biomarkers

**Figure 2:**
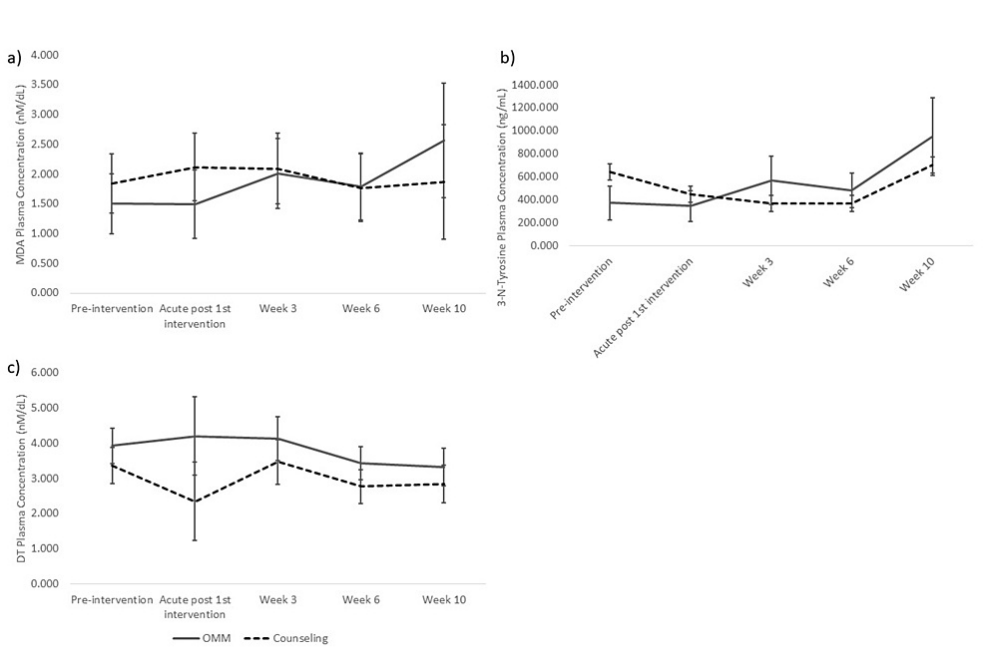
Efficacy of OMM in changing MDA, DT, NT-3 plasma levels. Plasma MDA means (± SEM) throughout and after a six-week intervention of OMM (n = 10) vs counseling (n = 9) in PD. p > 0.05. b) Plasma 3-N-Tyrosine means, OMM (n = 8) vs counseling (n = 3) in PD. p > 0.05. c) Plasma DT means, OMM (n = 5) vs counseling (n = 3) in PD. p > 0.05. OMM: Osteopathic Manipulative Medicine. MDA: Malondialdehyde. DT: L,L-dityrosine. 3-NT: 3-nitrotyrosine.

**Figure 3:**
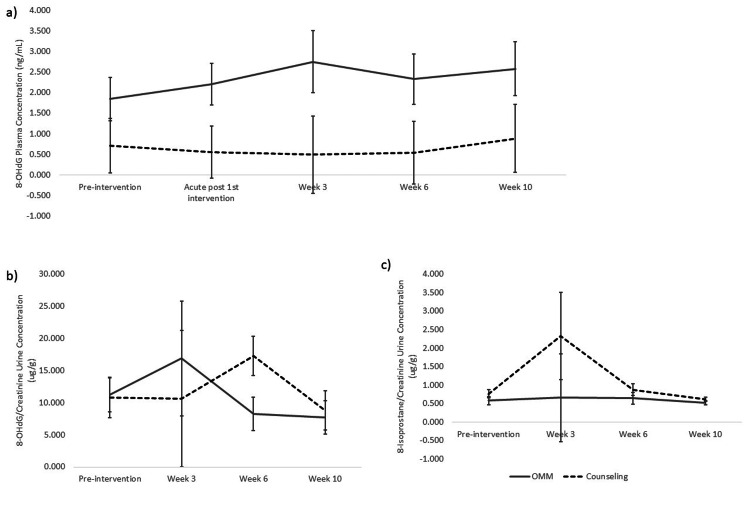
Efficacy of OMM in changing 8-OHdG plasma and 8-Isoprostane urine levels. Plasma 8-OHdG means (± SEM) throughout and after a 6-week intervention of OMM (n = 11) vs counseling (n = 7) in PD. p > 0.05. b) Urine 8-OHdG means (± SEM) normalized to urine creatinine, OMM (n = 7) vs counseling (n = 5) in PD. p > 0.05. c) Urine 8-isoprostane means, OMM (n = 4) vs counseling (n = 4) in PD. p > 0.05. OMM: Osteopathic Manipulative Medicine. 8-OHdG: 8-hydroxy-2-deoxyguanosine.

OMT on systemic MDA levels throughout and after 12 OMT sessions over six weeks were investigated (Figure 2a). No inter- or intragroup differences were found (p > 0.05). Following the same schedule, serum levels of 3-NT were assessed (Figure 2b). No inter- or intragroup differences were found (p> 0.05). Serum DT was also measured along the same course as above (Figure 2c). Similarly, no inter- or intragroup differences were found (p > 0.05). Serum and urinary 8-OHdG were determined following the same schedule as above (Figure 3a, 3b). No inter- or intragroup differences were found (p> 0.05). Urinary 8-isoprostane was measured as above (Figure 3c). Similarly, no inter- or intragroup differences were found (p > 0.05).

## Discussion

OMT has been shown to benefit individuals with PD as well as other older individuals suffering from postural and balance issues as well as subjects with dizziness and prone to falling [9-11]. In addition, there is evidence that OMT affects molecular biomarkers both acutely post-intervention and four weeks post-intervention [62-65]. Our current findings did not uncover any novel biomarker changes post-OMT, however subjects and caregivers reported verbally increased stability and ease of movement both of which need to be objectively assessed in future studies.

Here we show that OMT did not affect the measured biomarkers either acutely or chronically post-intervention. This may suggest that benefits seen after OMT treatments are not necessarily due to changes in oxidative stress mechanisms. Further research is required to elucidate the molecular effects of OMT in PD, perhaps investigating biomarkers specific for muscle activity, such as the PI-3K/Akt/mTOR pathway [66].

There are limited studies examining OMT and molecular biomarkers. The OMT protocol used for this study was focused on improving biomechanics, such as balance and gait as shown in previous studies, including stride length, cadence, upper and lower limb velocities, however biomarkers were not established for these studies [12,15]. It is important to point out that traditionally, osteopathic manipulations are completed exclusively for osteopathic lesions identified, however, in this research study, the authors always ensured equal treatment across all subjects.

While it is still unclear whether this treatment regimen affects production or clearance of ROS in any way, osteopathic lymphatic techniques have moved into focus and need to be investigated. Additionally, it must be considered that ROS vary greatly depending on many factors. Studies have shown that long-term diet, time of day, sleep, exercise, disease comorbidities, among others, all create variability in intra-subject ROS biomarkers, including MDA and DT [67-74].

For this study cohort, we did not account for or control these dynamics as this would have required unreasonable efforts by the study subjects. Subjects with PD also fluctuate greatly on a day-to-day basis as far as severity of symptoms, especially when skipping doses of their medication, which we asked to avoid due to their confounding nature [75]. The availability of caregivers for rides and severe weather situations, for example, resulted in treatments and measurements occurring at different hours of the day or missed visits altogether. The collective magnitude of the confounding effect remains unclear. Another common inconsistency is the fact that some PD subjects participate in exercise routines and classes designed to improve their symptoms. This could be an issue because physical exercise is known to positively impact systemic oxidative stress levels [45]. While we asked to not begin an exercise regimen while enrolled in the study, we did not exclude individuals already involved in such activities. To statistically control the exercise factor, we will need to quantify exercise time and intensity in future studies.

Foremost, the largest limitation we see is sample size. Unfortunately, recruitment was difficult (32 subjects over four years). We regularly attempted recruiting at local PD support groups, PD fundraisers, and through neurologists. Despite reaching our sample goal, many blood draws were unfortunately insufficient to run sufficient assays, further reducing the sample size.

When designing biomarker studies, one must consider the above confounding factors and attempt to control them. One of the seemingly easier factors to control is fasting state upon arrival. Subjects should be fasting when blood samples are extracted, however this does not account for longer-term/lifestyle diets. Additionally, due to longer measurement times (three to four hours), asking subject to fast for the morning and continue to not consume calories may be unreasonable. While the creation of a manageable testing environment is paramount, drawbacks in the form of variable outcomes need to be considered.

Time of day for treatment or measurement is another element that needs to be standardized. To overcome the above limitations, one could offer chaperoned transportation independent of the schedule of caregivers or the weather situation.

Sleep time and quality are, for the most part, beyond the control of the investigator, especially in subjects that are active and mobile. Sleep habits vary greatly on an individual basis and any attempted control would pose an unreasonable request interfering with the subject’s quality of life.

Accounting for disease comorbidities that might influence biomarker behavior is easily controllable through appropriate inclusion and exclusion criteria. However, in studies with focus on geriatric populations, limiting comorbidities to ensure similar overall health between subjects, could drastically impact recruiting efforts, diminish a study’s power, and render it impossible at a certain site altogether.

Despite significant research efforts, the diagnosis, management, and prognosis of PD have not fundamentally changed over the past few decades. The development of reliable predictive and outcome markers could revolutionize the field and point the way toward personal risk assessment and novel therapeutic strategies [76]. The role of oxidative stress in neuroinflammation and neurodegeneration has been heavily researched and reliable molecular biomarkers are beginning to emerge [22,77]. However, most new candidate biomarkers have only been studied in animal models and have yet to be rigorously tested in a clinical environment across various etiologies (e.g., genetic, idiopathic, drug-induced, environmental) and disease stages. It is becoming increasingly clear that new approaches are needed and currently the pursuit of combined biomarkers as well as the inclusion of machine learning and artificial intelligence to model disease states and their progression are at the forefront of interest [78,79]. These strategies will eventually yield novel tools for a more thorough and reliable assessment of the effect of OMT in PD.

## Conclusions

In summary, we were unable to provide conclusive support to our hypothesis that application of OMT may reduce the ROS burden seen in advanced-stage PD. The biomarkers we utilized are state-of-the-art for a variety of inflammatory and degenerative conditions tied to elevated systemic oxidative stress. However, it must be mentioned that the field of biomarker discovery is fast advancing with new combinatorial approaches (e.g., molecular and behavioral) on the horizon. Specific to PD via established biomarkers of oxidative stress. There were several limitations of this study, most importantly those potentially affecting highly reactive biomarkers. To better account for and offset the effects of controllable and non-controllable confounders, future OMT studies will require a more tightly controlled study environment and a larger sample size.
